# Exploring pta Alternatives in the Development of Ruthenium–Arene Anticancer Compounds

**DOI:** 10.3390/molecules28062499

**Published:** 2023-03-09

**Authors:** Jakob Kljun, Mihaela Rebernik, Lucía M. Balsa, Jerneja Kladnik, Uroš Rapuš, Tomaž Trobec, Kristina Sepčić, Robert Frangež, Ignacio E. León, Iztok Turel

**Affiliations:** 1 Faculty of Chemistry and Chemical Technology, University of Ljubljana, Večna pot 113, SI-1000 Ljubljana, Slovenia; 2 CEQUINOR (UNLP, CCT-CONICET La Plata, Asociado a CIC), Departamento de Química, Facultad de Ciencias Exactas, Universidad Nacional de La Plata, Blvd. 120 N°1465, La Plata 1900, Argentina; 3 Institute of Preclinical Sciences, Veterinary Faculty, University of Ljubljana, Gerbičeva 60, SI-1000 Ljubljana, Slovenia; 4 Department of Biology, Biotechnical Faculty, University of LjubljanaJamnikarjeva 101, SI-1000 Ljubljana, Slovenia

**Keywords:** ruthenium, pyrithione, phosphines, anticancer activity, glutathione S-transferase

## Abstract

Organoruthenium pyrithione (1-hydroxypyridine-2-thione) complexes have been shown in our recent studies to be a promising family of compounds for development of new anticancer drugs. The complex [(η^6^-*p*-cymene)Ru(pyrithionato)(pta)]PF_6_ contains phosphine ligand pta (1,3,5-triaza-7-phosphaadamantane) as a functionality that improves the stability of the complex and its aqueous solubility. Here, we report our efforts to find pta alternatives and discover new structural elements to improve the biological properties of ruthenium anticancer drugs. The pta ligand was replaced by a selection of phosphine, phosphite, and arsine ligands to identify new functionalities, leading to improvement in inhibitory potency towards enzyme glutathione *S*-transferase. In addition, cytotoxicity in breast, bone, and colon cancers was investigated.

## 1. Introduction

Ruthenium complexes have been studied extensively for their antitumor and antimetastatic properties over the past two decades. During these studies, several structural scaffolds have emerged on which researchers have based their investigations and development of improved analogues and derivatives. The best candidates have already been tested in clinical trials or studied in detail at the preclinical level [1,2,3,4,5]. In general, three major classes of compounds have emerged: (i) octahedral ruthenium(III)-chlorido complexes with *N*-heterocyclic ligands (such as clinical candidates NAMI-A and KP1339 [6]), (ii) photoactive polypyridyl complexes (such as clinical candidate TLD1433 [7]), and (iii) organoruthenium(II) compounds. Among the latter, the Dyson and Sadler groups developed what are now known as compound classes RAPTA and RAED [5,8]. The general structure of these compounds is [(η^6^-arene)(Ru)(chel)X]^n+^, in which the arene ligand is most commonly *p*-cymene, “chel” represents a bidentate chelating ligands (neutral or monoanionic), and X represents either halide leaving groups or more strongly bound phosphines, *N*-heterocycles, carbenes, or thiolato monodentate ligands. Among monodentate ligands, pta (1,3,5-triaza-7-phosphaadamantane) has played an important role due to the stability of its complexes and ability to increase aqueous solubility of the ruthenium species formed.

Among the different types of bidentate ligands, the most promising anticancer agents in terms of chemical stability, reactivity, binding to molecular targets (enzymes or DNA), and selective toxicity or antimetastatic activity were obtained by using *N,N*-ligands (diamines or aromatic diimines [9]), *N,O*-ligands (mainly hydroxyquinolines [10,11,12,13,14,15,16,17]), and *S,O*-ligands (thiopyrones, thiopyridones, pyrithiones [18,19,20,21,22,23,24,25,26]).

**Figure 1:**
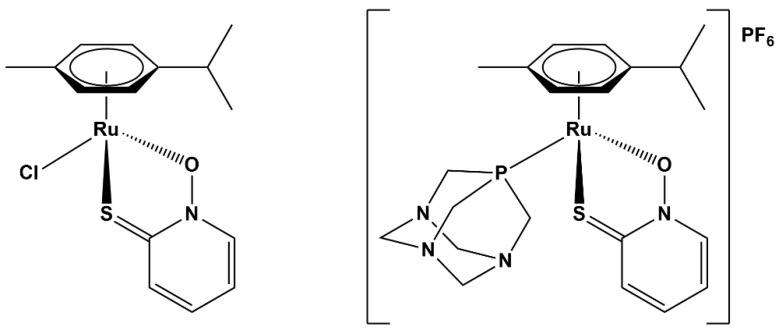
Parent organoruthenium–pyrithionato chlorido **1** and pta complex **2**.

The pyrithiones studied by our group have proven to be versatile ligands for development of bioactive ruthenium complexes, including parent compounds **1** [(η^6^-cymene)(Ru)(pyrithionato)Cl] and **2** [(η^6^-cymene)(Ru)(pyrithionato)(pta)]PF_6_ (Figure 1), which are capable of inhibiting several enzymatic targets related to cancer therapy, such as cathepsin B, glutathione *S*-transferase (GST), and aldo-keto reductases 1C (AKR1C1–3), with a generally favorable toxicity profile, with IC_50_ values for different cancer cell lines in the low micromolar range and virtually no toxic effect on healthy cells in the concentration ranges tested [22−24,26].

In addition, compound **1** was found to strongly inhibit cholinesterases, with virtually no adverse physiological responses to the neuromuscular system, which is a beneficial feature for potential anticancer drug candidates [25].

A set of analogs of ruthenium complexes **1** (chlorido complex) and **2** (pta complex) were extensively studied by our group in recent years as part of our effort to explore and identify structural elements in the pyrithionato scaffold to obtain potent and selective cytotoxic agents [22,23,24]. In general, most synthesized complexes exhibited IC_50_ values in the low micromolar range on most cancer cells tested (comparable to cisplatin) but with higher selectivity. Introduction of donor substituents on positions 3 and 5 of the pyrithione ring resulted in increased toxicity, with IC_50_ values descending to low-single-digit micromolar values. Furthermore, chlorido complexes bearing ligands with extended aromatic scaffolds (quinoline or isoquinoline analogs of pth) showed increased toxicity, while their pta analogs were mostly inactive at the concentration ranges tested. These chlorido complexes also showed remarkable ability to overcome platinum resistance in several ovarian cancer cell types [22].

**Figure 2:**
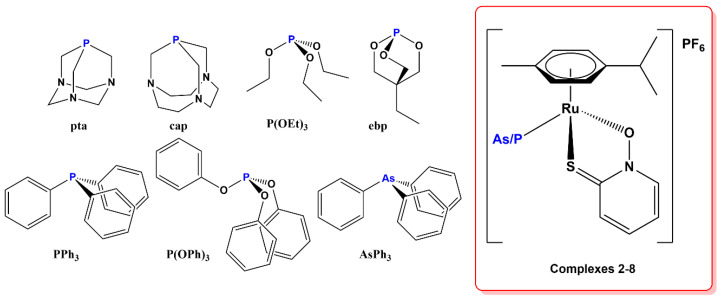
Phosphine, phosphite, and triphenyarsine ligands used in this study and the general structure of synthesized complexes **2**–**8**.

In this work, we investigate alternatives to pta as a monodentate ligand, introducing other commercially available or easily synthesized phosphine, phosphite, and triphenyarsine ligands. Among the six ligands selected were the larger pta analog cap (1,4,7-triaza-9-phosphatricyclo [5.3.2.1^4,9^]tridecane), triethylphosphite P(OEt)_3_, and bicyclic phosphite ebp (commercially available as ethyl bicyclic phosphite or 4-ethyl-2,6,7-trioxa-1-phosphabicyclo [2.2.2]octane), triphenylphosphine PPh_3_, and its analogs triphenylphosphite P(OPh)_3_ and triphenylarsine AsPh_3_ (Figure 2). Some of them have occasionally been used in ruthenium drug or catalyst development and were proven to form stable, water-soluble, and bioactive complexes, while the ruthenium coordination chemistry of others is mostly unexplored. Except for triethylphosphite and triphenylarsine, the literature mostly reports on the chemistry and catalytic properties of these complexes [27,28,29]. While all RAPTA-C analogues—i.e., dichloridoruthenium(*p*-cymene) complexes of selected P/As ligands—have been reported, only triphenylphosphine and cap have been studied for their anticancer effects. Phosphite complexes have been evaluated against alveolar echinococcosis, a disease caused by infection with the larval stage of tapeworm *Echinococcus multilocularis* [29]. RAPTA complexes with ligand cap were found to have higher toxicity compared to their pta analogues but no selectivity over healthy cells in the panel of cell lines tested [27]. Most importantly, Parveen et al. [30] performed a comparative study on the anticancer properties of organoruthenium–pyr(id)onato complexes containing either pta or triphenylphosphine. The phosphine ligands were used to stabilize the ruthenium–pyr(id)onato species, which otherwise undergo rapid hydrolysis to form biologically inactive species. They found that triphenylphosphine complexes exhibited much higher toxicity and attributed this to increased lipophilicity of the ligand [30].

Glutathione *S*-transferases are enzymes involved in the molecular mechanisms of biotransformation and detoxification of xenobiotic compounds [31,32]. They are overexpressed in breast, ovarian, colon, pancreatic, and other solid tumors [33]. Their increased activity can lead to degradation of anticancer drugs, particularly metallodrugs, which have a high affinity for thiol targets [34,35]; thus, GST inhibitors represent a promising class of new anticancer drugs. Parent complex **1** has been found to combine potent anticancer activity with GST inhibition [25].

We report here the synthesis and structural characterization of six new organoruthenium–pyrithionato complexes (**3**–**8**). All the new complexes and parent complexes **1** and **2** were studied as GST inhibitors, and their potential as anticancer agents was evaluated using bone, colon, and breast cancer cells.

## 2. Results and Discussion

### 2.1. Synthesis and Characterization

**Figure 3:**
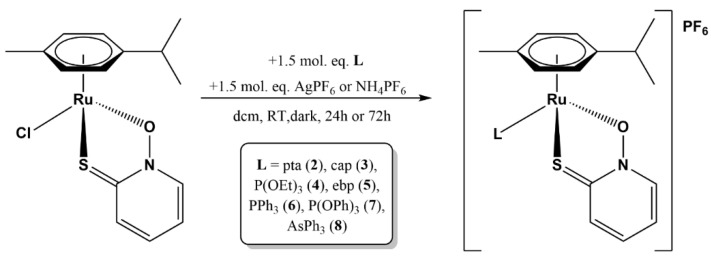
General reaction scheme for preparation of ruthenium–pyrithionato complexes **2**–**8** with different phosphine, phosphite, or arsine ligands.

Complexes **1** and **2** were synthesized according to previously described procedures [24,26]. Complexes **3**–**8** were synthesized by reacting chlorido complex **1** with 1.5 molar equivalents of the selected phosphine (pta, cap, PPh_3_), phosphite (ebp, P(OEt)_3_, P(OPh)_3_), or triphenylarsine (AsPh_3_) ligand in the presence of ammonium or silver hexafluorophosphate salts, which are used to facilitate chloride dissociation (Figure 3). The reactions were carried out in Na_2_SO_4_-dried dichloromethane or chloroform over a period of 24–72 h. Formation of a fine precipitate was observed, and the color of the reaction mixture changed from deep orange to yellow. If the reaction mixture contained silver salts, the reactions were carried out in the dark to avoid formation of elemental silver. 

Thin-layer chromatography on silica was used to monitor the reaction rate. The neutral starting ruthenium complex generally had a much higher retention factor than cationic complexes **2**–**7** when 2–10% methanol in dichlorometane (DCM) or chloroform was used as the mobile phase. The reaction mixtures were filtered through a thin layer of celite to remove the precipitated inorganic salts, and the remaining solutions were concentrated to 1–2 mL and purified by column chromatography on silica using the same mobile phases. Occasionally, the methanol percentage was slightly increased once the desired complex began to elute to speed up the elution. Precipitation from a concentrated oily solution was achieved by adding cold diethyl ether and/or *n*-heptane, while precipitations with hexane(s) and petrol ethers were less successful, occasionally yielding oily products. The complexes are well soluble in chloroform, DCM, acetone, dmso, dmf, and methanol, sparingly soluble in ethanol and water (maximum solubility about 1 mg/mL), and insoluble in diethyl ether and alkanes.

**Figure 4:**
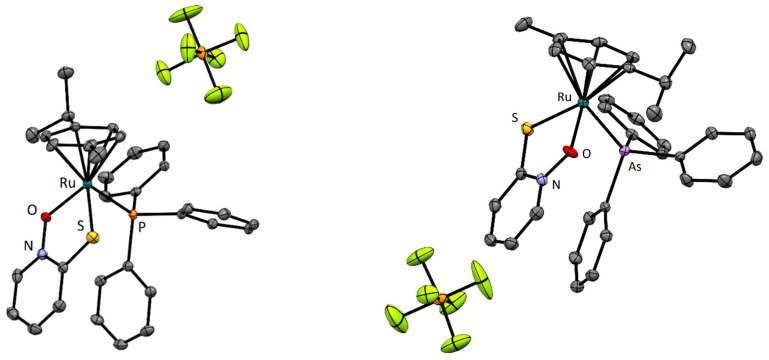
Crystal structures of complexes **6** (**left**) and **8** (**right**). Thermal ellipsoids were drawn at 35% probability level, and hydrogen atoms were omitted.

We succeeded in obtaining high-quality crystals of compounds **6** and **8**. The crystal structures show the expected pseudooctahedral geometry in which the phosphine/arsine ligand replaces the chlorido ligand from parent compound **1**. Due to the absence of hydrogen bond donors, the structure lacks strong intermolecular interactions. The structure is stabilized by weak C_Ar_–H…F interactions, with hexafluorophosphate anion and π-stacking interactions between the pyrithionato ring and one of the phenyl groups of the triphehylphosphine/triphenylarsine ligand. All bond lengths and angles are within the values reported for structurally similar organoruthenium complexes (Figure 4, Table 1).

### 2.2. Cytotoxicity Studies

Cell viability studies were determined by the MTT assay for complexes **1**–**8** toward MCF7 (breast adenocarcinoma), MDA-MB-231 (triple-negative breast adenocarcinoma), MG-63 (osteosarcoma), and HT-29 (colorectal carcinoma) after 24 h of incubation with new ruthenium compounds. Cisplatin was used as antitumor clinical reference. The three solid tumors selected for the cell viability study were chosen due to high incidence and the low efficacy of the current treatments. The two breast cancer cell lines used in this study represent two of the most relevant subtypes of breast cancer (MCF7: receptor-estrogen-positive breast cancer subtype; MDA-MB-231: triple-negative breast cancer subtype).

As can be seen in Table 2, complex **6** showed good anticancer activity against breast, bone, and colorectal cancer cells, whilst complex **7** did not exert anticancer effect on colorectal cancer cells. The IC_50_ values indicate that compound **6** exerts stronger antitumor effects than **7**. Moreover, compound **8** impaired the cell viability of all the cancer cell lines tested, showing moderate anticancer activity (IC_50_ = 35–50 µM), and compound **4** only showed anticancer effects with moderate activity (IC_50_ = 45 µM) on MCF7 cells. Finally, compounds **1–3** and **5** showed relatively low activity, with IC_50_ higher than 50 µM in all cell lines tested.

On the other hand, it is important to highlight that compounds **6** and **7** are more active than cisplatin in all the tested cell lines. For compound **6,** an IC_50_ value was six-fold lower on HT-29 cells and twelve-fold lower on MDA-MB-231 cells than in the case of cisplatin. Furthermore, comparison with our previous studies on ruthenium–hydroxyquinolinato complexes shows that complex **1** has much lower toxicity than its hydroxyquinolinato counterparts (IC_50_ values in the concentration range 8–50 µM for halido complexes), but toxicity increases again to similar levels when lipophilic phosphine and arsine ligands are included [16,36].

These findings are in line with the results of the Hartinger group [30], where the appropriate choice of monodentate ligand resulted in stabilization of the ruthenium species. Furthermore, the increase in general toxicity can be partially attributed to the increased lipophilicity of the monodentate ligand as the three most toxic compounds all possess three phenyl rings on the respective monodentate ligands (PPh_3_, P(OPh)_3_, and AsPh_3_). 

Among the cell lines tested, the MDA-MB-231 (triple-negative breast adenocarcinoma) has particularly elevated levels of GST, which usually results in drug resistance [37]. These cell lines were also proven to be the most sensitive to the compounds tested.

### 2.3. GST Inhibition

**Figure 5:**
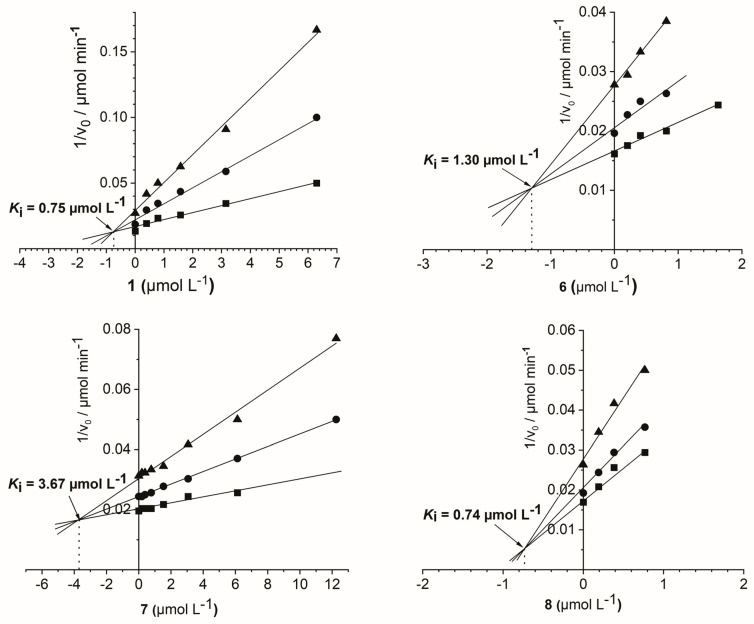
Dixon plots for determination of the type of inhibition and inhibition constants (*K_i_*) for compounds **1**, **6**, **7**, and **8** towards glutathione *S*-transferase from horse liver (GST). The concentrations of the substrate 1-chloro-2,4-dinitrobenzene were 200 μM (▲), 400 μM (●), and 800 μM (■).

In the present study, eight ruthenium compounds were tested for their inhibitory activity towards GST, and the inhibition parameters (IC_50_, *K_i_*) are shown in Table 3. Five of these compounds (**1**, **4**, **6**–**8**) showed inhibitory activities at low micromolar range. Significant inhibition of GST in a pharmaceutically interesting range was shown by compounds **1**, **6, 7,** and **8**, exhibiting IC_50_ values of 3.15, 11.84, 2.88, and 2.15 µM, respectively. The inhibition was of reversible competitive type in all cases, with *K_i_* values in low micromolar or even in submicromolar range, as shown in Table 3 and Figure 5. The GST active site comprises H-site, which is involved in accommodation of a variety of hydrophobic substrates, and G-site, which binds glutathione and enables nucleophilic attack of the glutathione thiol group upon the substrate [38]. The inhibition of GST obtained with the compounds tested in this study might derive from their competitive binding to the H-site due to its much lower specificity [39]. A similar mechanism of GST inhibition was proposed for (η^6^-*p*-cymene)ruthenium-based compounds studied by Ang et al. [34,35].

## 3. Materials and Methods

### 3.1. Synthesis and Characterization

Complexes **1**, **2,** and the ligand cap (1,4,7-triaza-9-phosphatricyclo [5.3.2.1^4,9^]tridecane) were synthesized according to published procedures [24,26,40]. All other ligands and solvents were purchased from Sigma-Aldrich except for ligand ebp (4-ethyl-1-phospha-2,6,7-trioxabicyclo [2.2.2]octane), which was purchased from TCI. All chemicals were used without purification or drying unless specified in the reported synthetic protocols. Reaction schemes are included in the Supporting Information File (Figures S1–S6). Physicochemical characterization of the prepared compounds was performed by ^1^H and ^31^P NMR spectroscopy (Figures S7–S13), CHN elemental analysis, high-resolution electrospray ionization mass spectrometry (ESI-HRMS), infrared (IR), UV–vis spectroscopy, and in two cases also with X-ray diffraction on single crystal. ^1^H and ^31^P NMR spectra were recorded using a Bruker Avance III 500 spectrometer at room temperature and 500 MHz and 202 MHz, respectively. The chemical shifts of the ^1^H NMR spectra are referenced to the deuterated residual solvent peaks of CDCl_3_ at 7.26 ppm. The splitting of the proton resonances is defined as s = singlet, d = doublet, t = triplet, q = quartet, hept = heptet, m = multiplet, and bs = broad signal. Chemical shift (*δ*) is given in ppm and coupling constants (*J*) in Hz. NMR data processing was carried out with MestReNova version 11.0.4. Figures of NMR spectra are included in the Supporting Information File (Figures S1–S13). Infrared spectra were recorded with a Bruker FTIR Alpha Platinum ATR spectrometer and high-resolution mass spectra (HRMS) on an Agilent 6224 Accurate Mass TOF LC/MS instrument. Elemental analyses were performed on a Perkin-Elmer 2400 II instrument (C, H, N). UV–vis spectra for compounds were obtained on PerkinElmer LAMBDA 750 UV/vis/near-IR spectrophotometer.

#### 3.1.1. [(η^6^-*p*-cym)Ru(κ^2^-O,S-C_5_H_4_NOS)(cap)]PF_6_ (**3**)

Complex **1** (50 mg; 0.126 mmol), ligand cap (37.0 mg; 0.189 mmol; 1.5 mol. eq.), and AgPF_6_ (47.6 mg; 0.189 mmol; 1.5 mol. eq.) were dissolved in 20 mL of dry dichloromethane in a 50 mL round-bottom flask. The reaction mixture was stirred in the dark at room temperature for 24 h. During this time, TLC was performed on silica with 5% MeOH/CHCl_3_ as the mobile phase to confirm the quantitative formation of the product and the absence of the ruthenium starting compound. The resulting AgCl precipitate was filtered over celite, the volume of the reaction mixture was reduced to 1–2 mL, and the product was purified by column chromatography using silica as the stationary phase. The product was eluted with a 7% MeOH/DCM mixture. The solvent was removed and the oily residue dissolved in a minimal amount of DCM and precipitated with diethyl ether. The pale-yellow powder was dried overnight at 45 °C (m = 83.3 mg, η = 93.7%).

**^1^H NMR** (500 MHz, CDCl_3_): δ 8.01–8.00 (d, 1H, Ar-*H* pth), 7.49–7.47 (d, 1H, Ar*-H* pth), 7.18 (t, 1H, Ar-*H* pth), 6.91–6.89 (t, 1H, Ar-*H* pth), 6.01–5.95 (m, 2H, Ar-*H* cym), 5.71–5.70 (m, 1H, Ar-*H* cym), 5.61–5.61 (m, 1H, Ar-*H* cym), 3.49–3.31 (m, 6H, cap), 3.02–2.91 (m, 12H, cap), 2.58 (m, 1H, Ar-C*H*(CH_3_)_2_), 2.08 (s, 3H, Ar-C*H*_3_), 1.25–1.20 (m, 6H, Ar-CH(C*H*_3_)_2_).

**^31^P NMR** (202 MHz, CDCl_3_): δ 56.84, −137.31, −141.04, −144.19, −147.76, −151.12.

**IR** (cm^−1^, ATR): 2975, 2866, 2810, 1647, 1442, 1404, 1375, 1355, 1310, 1289, 1256, 1241, 1198, 1143, 1051, 1029, 945, 897, 838, 740, 657, 624.

**HRMS** (*m*/*z*): [M-PF_6_^−^]^+^ C_24_H_36_N_4_OPRuS^+^ found 561.1388 (calculated 561.1391).

**CHN:** found C 40.51; H 5.19; N 7.75; calculated C 40.85; H 5.14; N 7.94. 

#### 3.1.2. [(η^6^-*p*-cym)Ru(κ^2^-O,S-C_5_H_4_NOS)(P(OEt)_3_)]PF_6_ (**4**)

Complex **1** (50 mg; 0.126 mmol), ligand triethylphosphite (P(OEt)_3_; 32.4 µL; 0.189 mmol; 1,5 mol. eq.), and NH_4_PF_6_ (30.8 mg; 0.189 mmol; 1.5 mol. eq.) were dissolved in 20 mL dry dichloromethane in a 50 mL round-bottom flask. The reaction mixture was stirred at room temperature for 24 h. During this time, TLC was performed on silica with 5% MeOH/CHCl_3_ as the mobile phase to confirm the quantitative formation of the product and the absence of the ruthenium starting compound. The resulting NH_4_Cl precipitate was filtered over celite, the volume of the reaction mixture was reduced to 1–2 mL, and the product was purified by column chromatography using silica as the stationary phase. The product was eluted with a 10% MeOH/DCM mixture. The solvent was removed and the oily residue dissolved in a minimal amount of DCM and precipitated with diethyl ether. The pale-yellow powder was dried overnight at 45 °C (m = 34.2 mg, η = 40.3%).

**^1^H NMR** (500 MHz, CDCl_3_): δ 8.09 (dd, 1H, Ar-H pth), 7.48 (dd, 1H, Ar-H pth), 7.20 (ddd, 1H, Ar-H pth), 6.93 (td, 1H, Ar-H pth), 5.92 (dd, 1H, Ar-H cym), 5.72 (qd, 2H, Ar-H cym), 5.65 (dd, 1H, Ar-H cym), 3.99 (pd, 6H, phosphite ligand), 2.75 (hept, 1H, Ar-C*H*(CH_3_)_2_), 2.24 (s, 3H, Ar-CH_3_), 1.30–1.15 (m, 15H, Ar-CH(C*H*_3_)_2_, phosphite ligand).

**^31^P NMR** (202 MHz, CDCl_3_): δ 87.87, −137.29, −140.81, −144.33, −147.84, −151.36.

**IR** (cm^−1^, ATR): 3099, 2979, 1600, 1547, 1461, 1413, 1389, 1263, 1243, 1158, 1136, 1092, 1044, 1015, 953, 877, 830, 782, 763, 722, 708, 682, 623.

**HRMS** (*m*/*z*): [M-PF_6_^−^]^+^ C_21_H_33_NO_4_PRuS^+^ found 528.0914 (calculated 528.0911).

**CHN:** found C 37.43; H 4.96; N 2.10; calculated C 37.50; H 4.95; N 2.08. 

#### 3.1.3. [(η^6^-*p*-cym)Ru(κ^2^-O,S-C_5_H_4_NOS)(epb)]PF_6_ (**5**)

Complex **1** (50 mg; 0.126 mmol), ligand ebp (30.6 mg; 0.189 mmol; 1,5 mol. eq.), and AgPF_6_ (47.6 mg; 0.189 mmol; 1.5 mol. eq.) were dissolved in 20 mL dry dichloromethane in a 50 mL round-bottom flask. The reaction mixture was stirred in the dark at room temperature for 24 h. During this time, TLC was performed on silica with 5% MeOH/CHCl_3_ as the mobile phase to confirm the quantitative formation of the product and the absence of the ruthenium starting compound. The resulting AgCl precipitate was filtered over celite, the volume of the reaction mixture was reduced to 1–2 mL, and the product was purified by column chromatography using silica as the stationary phase. The product was eluted with a 10% MeOH/DCM mixture. The solvent was removed and the oily residue dissolved in a minimal amount of DCM and precipitated with diethyl ether. The pale yellow powder was dried overnight at 45 °C (m = 33.7 mg, η = 40.0%).

**^1^H NMR** (500 MHz, CDCl_3_): δ 8.03 (dd, 1H, Ar*-H* pth), 7.48 (dd, 1H, Ar-*H* pth), 7.22 (ddd, 1H, Ar-*H* pth), 6.92 (td, 1H, Ar-*H* pth), 6.02 (dd, 1H, Ar-*H* cym), 5.89 (dd, 1H, Ar-*H* cym), 5.86–5.82 (m, 1H, Ar*-H* cym), 5.68 (dd, 1H, Ar-*H* cym), 4.32 (d, 6H, ebp), 2.73 (hept, 1H, Ar-C*H*(CH_3_)_2_), 2.21 (s, 3H, Ar-C*H*_3_), 1.35–1.22 (m, 8H, Ar-CH(C*H*_3_)_2_, ebp) 0.82 (t, 3H, ebp).

**^31^P NMR** (202 MHz, CDCl_3_): δ 85.82, −137.25, −140.77, −144.29, −147.81, −151.33.

**IR** (cm^−1^, ATR): 2975, 2896, 1600, 1552, 1460, 1413, 1392, 1245, 1151, 1135, 1028, 952, 939, 876, 832, 811, 784, 752, 710, 681, 644.

**HRMS** (*m/z*): [M-PF_6_^−^]^+^ C_21_H_29_NO_4_PRuS^+^ found 524.0605 (calculated 524.0598).

**CHN:** found C 36.68; H 4.21; N 2.18; calculated C 37.73; H 4.37; N 2.10. 

#### 3.1.4. [(η^6^-*p*-cym)Ru(κ^2^-O,S-C_5_H_4_NOS)(PPh_3_)]PF_6_ (**6**)

Complex **1** (50 mg; 0.126 mmol), ligand triphenylphosphine (PPh_3_; 49.6 mg; 0.189 mmol; 1.5 mol. eq.), and AgPF_6_ (47.6 mg; 0.189 mmol; 1.5 mol. eq.) were dissolved in 20 mL of dry dichloromethane in a 50 mL round-bottom flask. The reaction mixture was stirred in the dark at room temperature for 72 h. During this time, TLC was performed on silica with 5% MeOH/CHCl_3_ as the mobile phase to confirm the quantitative formation of the product and the absence of the ruthenium starting compound. The resulting AgCl precipitate was filtered over celite, the volume of the reaction mixture was reduced to 1–2 mL, and the product was purified by column chromatography using silica as the stationary phase. The product was eluted with a 2% MeOH/DCM mixture. The solvent was removed and the oily residue dissolved in a minimal amount of DCM and precipitated with diethyl ether. The pale yellow powder was dried overnight at 45 °C (m = 64.9 mg, η = 85.2%). Crystals suitable for X-ray diffraction were obtained with diethyl ether vapor diffusion into a concentrated DCM solution of **6**.

**^1^H NMR** (500 MHz, CDCl_3_): δ 7.64 (ddd, 1H, Ar-*H* pth), 7.53–7.45 (m, 6H, Ar-*H* PPh_3_), 7.45–7.31 (m, 9H, Ar-*H* PPh_3_), 7.03 (ddd, 1H, Ar-*H* pth), 6.87 (ddd, 1H, Ar-*H* pth), 6.58 (td, 1H, Ar-*H* pth), 5.92 (dd, 1H, Ar-*H* cym), 5.64 (dd, 1H, Ar-*H* cym), 5.32–5.26 (m, 1H, Ar-*H* cym), 5.22 (dd, 1H, Ar-*H* cym), 2.65 (hept, 1H, Ar-C*H*(CH_3_)_2_), 1.78 (s, 3H, Ar-C*H*_3_), 1.28–1.16 (m, 3H, Ar-CH(C*H*_3_)_2_), 1.09 (d, 3H, Ar-CH(C*H*_3_)_2_).

**^31^P NMR** (202 MHz, CDCl_3_): δ 35.35, −137.25, −140.77, −144.29, −147.81, −151.33.

**IR** (cm^−1^, ATR): 2973, 1550, 1480, 1458, 1434, 1242, 1138, 1095, 1053, 830, 759, 744, 710, 697, 627.

**HRMS** (*m/z*): [M-PF6^−^]^+^ C_33_H_33_NOPRuS^+^ found 624.1063 (calculated 624.1064).

**CHN:** found C 51.23; H 4.35; N 1.77; calculated C 51.56; H 4.33; N 1.82. 

#### 3.1.5. [(η^6^-*p*-cym)Ru(κ^2^-O,S-C_5_H_4_NOS)(P(OPh)_3_)]PF_6_ (**7**)

Complex **1** (50 mg; 0.126 mmol), ligand triphenylphosphite (P(OPh)_3_; 59,9 mg; 0.186 mmol; 1.5 mol. eq.), and NH_4_PF_6_ (30,8 mg; 0.189 mmol; 1.5 mol. eq.) were dissolved in 20 mL of dry dichloromethane in a 50 mL round-bottom flask. The reaction mixture was stirred at room temperature for 24 h. During this time, TLC was performed on silica with 5% MeOH/CHCl_3_ as the mobile phase to confirm the quantitative formation of the product and the absence of the ruthenium starting compound. The resulting NH_4_Cl precipitate was filtered over celite, the volume of the reaction mixture was reduced to 1–2 mL, and the product was purified by column chromatography using silica as the stationary phase. The product was eluted with a 7% MeOH/DCM mixture. The solvent was removed, the oily residue dissolved in a minimal amount of CHCl_3_, and precipitated using 1:1 mixture of diethyl ether and heptane. The pale yellow powder was dried overnight at 45 °C (m = 35.9 mg, η = 42.7%). 

**^1^H NMR** (500 MHz, CDCl_3_) δ 7.97 (dd, *J* = 6.7, 1.2 Hz, 1H, Ar-*H* pth), 7.42 (dd, *J* = 8.4, 1.7 Hz, 1H, Ar-*H* pth), 7.39–7.27 (m, 6H, P(OPh)_3_), 7.16 (m, 4H, 1 Ar-*H* pth and 3 P(OPh)_3_), 7.09 (d, *J* = 7.1 Hz, 6H, P(OPh)_3_), 6.87 (td, *J* = 7.0, 1.7 Hz, 1H, Ar-*H* pth), 5.91 (dd, *J* = 6.4, 1.5 Hz, 1H, Ar-*H* cym), 5.59 (dd, *J* = 6.4, 1.5 Hz, 1H, Ar-*H* cym), 5.41 (dd, *J* = 6.3, 1.5 Hz, 1H, Ar-*H* cym), 5.24 (dd, *J* = 6.4, 1.4 Hz, 1H, Ar-*H* cym), 2.22 (hept, *J* = 6,9 Hz, 1H, Ar-C*H*(CH_3_)_2_ cym), 1.81 (s, 3H, Ar-C*H*_3_ cym), 1.14 (d, *J* = 6.9 Hz, 6H, Ar-CH(C*H*_3_)_2_ cym).

**^31^P NMR** (202 MHz, CDCl_3_) δ 111.63, −137.25, −140.77, −144.29, −147.81, −151.33.

**HRMS** (*m*/*z*): [M-PF_6_^−^]^+^ C_33_H_33_NO_4_PRuS^+^ found 672.0924 (calculated 672.0920).

**CHN:** found C 47.99; H 4.02; N 2.01; calculated C 48.53; H 4.07; N 1.72. 

#### 3.1.6. [(η^6^-*p*-cym)Ru(κ^2^-O,S-C_5_H_4_NOS)(AsPh_3_)]PF_6_ (**8**)

Complex **1** (50 mg; 0.126 mmol), ligand triphenylarsine (AsPh_3_; 56,9 mg; 0.186 mmol; 1.5 mol. eq.), and AgPF_6_ (47.6 mg; 0.189 mmol; 1.5 mol. eq.) were dissolved in 20 mL of dry dichloromethane in a 50 mL round-bottom flask. The reaction mixture was stirred in the dark at room temperature for 24 h. During this time, TLC was performed on silica with 5% MeOH/CHCl_3_ as the mobile phase to confirm the quantitative formation of the product and the absence of the ruthenium starting compound. The resulting AgCl precipitate was filtered over celite, the volume of the reaction mixture was reduced to 1–2 mL, and the product was purified by column chromatography using silica as the stationary phase. The product was eluted using a 5% and 10% MeOH/CHCl_3_ mixture. The solvent was removed, the oily residue dissolved in a minimal amount of CHCl_3_, and precipitated using 1:1 mixture of diethyl ether and heptane. The pale yellow powder was dried overnight at 45 °C (m = 35.9 mg, η = 42.7%). Crystals suitable for X-ray diffraction were obtained from a concentrated DCM/hexane solution of **8** at 5 °C overnight0.

**^1^H NMR** (500 MHz, CDCl3) δ 7.68 (d, J = 6.8 Hz, 1H, Ar-*H* pth), 7.42–7.34 (m, 15H, Ar-*H* AsPh_3_), 7.03 (d, 1H, Ar-*H* pth), 6.86 (t, J = 7.7 Hz, 1H, Ar-*H* pth), 6.58 (td, J = 6.9, 1.7 Hz, 1H, Ar-*H* pth), 5.92–5.66 (m, 2H, Ar-*H* cym), 5.47 (d, J = 6.0 Hz, 2H, Ar-*H* cym), 2.62 (p, J = 6.9 Hz, 1H, Ar-C*H*(CH_3_)_2_ cyl), 1.88 (s, 3H, Ar-C*H*_3_ cym), 1.15 (d, J = 6.5 Hz, 6H, Ar-CH)C*H*_3_)_2_).

**HRMS** (*m*/*z*): [M-AsPh_3_-PF_6_]^+^ C_15_H_18_NORuS^+^ found 362.0145 (calculated 362.0153)

**CHN**: found C: 49.23; H: 3.96; N: 1.79; calculated C: 48.77; H: 4.09; N: 1.72.

### 3.2. Crystallography

X-ray diffraction data were collected using an Oxford Diffraction SuperNova diffractometer with a Mo/Cu microfocus X-ray source (K_α_ radiation, λ_Mo_ = 0.71073 Å, λ_Cu_ = 1.54184 Å) with mirror optics and an Atlas detector at 150(2) K. Structures were solved in Olex^2^ graphical user interface [41] by direct methods implemented in SHELXT and refined by a full-matrix least-squares procedure based on F^2^ using SHELXL [42]. All non-hydrogen atoms were refined anisotropically. Hydrogen atoms were placed at calculated positions and treated using appropriate riding models. The crystal structure has been submitted to CCDC and has been assigned the deposition numbers 2235957–2235958. Additional crystallographic data (Table S1) and photographs of the analyzed crystals (Figure S14) are included in the Supplementary information file.

### 3.3. Cell Line and Growth Conditions

Human breast cancer cell lines (MCF-7 and MDA-MB-231), human bone cancer cell line (MG-63), and human colorectal cancer cell line (HT-29) were grown in Dulbecco’s modified Eagle’s medium (DMEM) containing 10% fetal bovine serum (FBS), 100 IU/mL penicillin, and 100 μg/mL streptomycin at 37 °C in 5% CO_2_ atmosphere. These cancer cell lines were grown in a 75 mL flask until they reached 70–80% confluence. Then, the cells were subcultured using TrypLE TM. For experiments, cells were grown in multi-well plates. Dulbecco’s modified Eagle’s medium (DMEM) and TrypLE^TM^ were purchased from Gibco (Gaithersburg, MD, USA), and fetal bovine serum (FBS) was purchased from Internegocios (Argentina). After 24 h, the monolayers were washed with DMEM and incubated under different conditions according to the experiments. Tissue culture materials were purchased from Corning (Princeton, NJ, USA). 

### 3.4. Cell Viability: 3-(4,5-Dimethylthiazol-2-yl)-2,5-Diphenyltetrazolium Bromide Assay 

The 3-(4,5-dimethylthiazol-2-yl)-2,5-diphenyltetrazolium bromide (MTT) assay was performed according to Mosmann [43]. Briefly, cells were seeded in a 96-well dish, allowed to attach for 24 h, and treated with different concentrations of complexes 4 to 8 (2.5–100 µM) at 37 °C for 24 h. Afterward, the medium was changed and the cells were incubated with 0.5 mg/mL MTT under normal culture conditions for 3 h. Cell viability was marked by conversion of the tetrazolium salt MTT to a colored formazan by mitochondrial dehydrogenases. Color development was measured spectrophotometrically with a microplate reader multiskan FC (Thermo scientific) at 570 nm after cell lysis in DMSO (100 µL per well). Cell viability was plotted as the percentage of the control value.

### 3.5. GST Inhibition Assay

The activity of GST was measured according to the method described by Habig et al. (1974) [44]. Stock solutions of **1**–**8** (1 mg/mL) were prepared in 100% methanol (MeOH). Then, stock solutions of **1**–**8** and negative controls were added to the wells and progressively diluted in 100 mM sodium phosphate buffer (pH 6.5) at a final volume of 50 μL. 1-Chloro-2,4-dinitrobenzene (Sigma-Aldrich Co., St. Louis, MO, USA) was dissolved in EtOH to obtain a 50 mM solution, then diluted with 100 mM sodium phosphate buffer (pH 6.5) to a final concentration of 4 mM. This solution (50 μL) and solution of 2 mM reduced glutathione (100 μL), dissolved in the same buffer were added into the microtiter plate wells. Glutathione *S*-transferase from horse liver (GST; Sigma-Aldrich Co., St. Louis, MO, USA) was used as the source of enzyme and dissolved in 100 mM sodium phosphate buffer (pH 6.5). 50 μL of enzyme solution was added into wells to start the reaction. The final enzyme concentration was 0.044 U/mL. A blank reaction was performed with the appropriate dilution of MeOH. The reaction was followed spectrophotometrically at 340 nm and at 25 °C for 4 min using a Cytation 3 cell imaging multi-mode reader from BioTek (BioTek, Winooski, VT, USA). Each measurement was repeated at least three times. For determination of inhibitory constants (*K_i_*), the kinetics were monitored using three different final substrate concentrations (200, 400, and 800 μM, respectively).

## 4. Conclusions

In this study, we have investigated possible alternatives to phosphine ligand pta in development of new organoruthenium(II) complexes with potential antitumor properties. Using six different commercially available or easily prepared phosphine, phosphite, or arsine ligands as monodentate ligands binding to the ruthenium(*p*-cymene)-pirithionato core, we prepared a series of new ruthenium compounds. Two crystal structures were determined and confirmed the expected pseudo-octahedral geometry of the synthesized complexes. The synthesized complexes were tested as glutathione *S*-transferase inhibitors and evaluated for their anticancer activity against a range of cancer cell lines, including breast, bone, and colon cancers. Complexes **6**–**8** with the most lipophilic ligands with three phenyl rings in the structure of the monodentate ligand (PPh_3_, P(OPh)_3_, and AsPh_3_) showed the strongest inhibition of GST, with Ki values in the low micromolar and submicromolar range, and the strongest toxic effect on selected breast, colon, and bone cancer cells. This effect was particularly pronounced in triple-negative breast cancer adenocarcinoma, which has elevated GST levels, which, in this case, may indicate a relationship between GST inhibition and toxicity. Our studies also show that, although substitution of a chlorido ligand for pta results in chemically less reactive species (and thus species with higher stability), this often results in reduced activity toward selected molecular targets and lower toxicity. However, this effect is reversed when lipophilic triphenylphosphine/phosphite/arsine ligands are used instead of pta. These complexes are ideally suited for further investigation in in vivo experiments, especially for the study of new breast cancer treatments.

## Figures and Tables

**Table 1:** Selected bond lengths in ruthenium–pyrithionato complexes (Å).

Compound	Ru-O	Ru-S	Ru-Cl/P/As
**1** [26]	2.081(2)	2.3555(7)	2.4488(7)
**2** [24]	2.075(2)	2.3490(7)	2.2806(6)
**6**	2.096(2)	2.3488(9)	2.352(1)
**8**	2.093(2)	2.3575(9)	2.4634(5)

**Table 2:** IC_50_ (µM) ± SD values of **1**–**8** and CDDP on MCF7, MDA-MB-231, MG-63, and HT-29 cell lines after 24 h of incubation.

	MCF7	MDA-MB-231	MG-63	HT-29
**1**	>100	>100	>100	>100
**2**	>100	>100	>100	>100
**3**	>100	>100	>100	>100
**4**	45.5 ± 2.4	>100	>100	>100
**5**	>100	>100	>100	>100
**6**	21.2 ± 3.2	10.8 ± 1.0	21.2 ± 1.7	30.2 ± 1.4
**7**	33.7 ± 6.3	18.0 ± 1.7	28.2 ± 2.5	>100
**8**	35.3 ± 12.1	35.8 ± 11.4	50.3 ± 2.5	40.8 ± 8.5
**Cisplatin**	42 ± 3.2	131 ± 18	39 ± 1.8	180 ± 6.0

**Table 3:** Inhibition of glutathione *S*-transferase from horse liver (GST) by ruthenium compounds tested.

Compound	GST	
No.	IC_50_ (µM)	*K_i_* (µM)
**1**	3.15 ± 2.5	0.75
**2**	/	*n. d.*
**3**	/	*n. d.*
**4**	59.49 ± 0.9	*n. d*.
**5**	/	*n. d.*
**6**	11.84 ± 1.1	1.30
**7**	2.88 ± 0.9	3.67
**8**	2.15 ± 0.4	0.74

IC_50_: concentration of the compound inducing 50% inhibition of the enzyme activity. *K_i_*: inhibitory constant; /: no inhibitory activity in tested concentration range (<125 µg/mL); *n. d.*: non-determined. *K_i_* values were not determined for compounds with IC_50_ > 20 µM.

## Data Availability

The data presented in this study are available in this article.

## Supplementary Materials

The following supporting information can be downloaded at: https://www.mdpi.com/article/10.3390/molecules28062499/s1, Figures S1–S6: reaction schemes; Figures S7–S13: NMR spectra of the compounds; Figure S14: photographs of the analyzed single crystals; Table S1: Crystallographic data table.

## Sample Availability

Samples of the compounds are available from the authors upon request.
