# Supplementary material for: Exploring pta Alternatives in the Development of Ruthenium–Arene Anticancer Compounds
Source: Molecules. 2023 Mar 9;28(6):2499. doi: 10.3390/molecules28062499 (PMC10058425; doi:10.3390/molecules28062499)

Exploring pta Alternatives in the Development of Ruthenium-Arene Anticancer Compounds

Jakob Kljun [1,*], Mihaela Rebernik [1], Lucia M. Balsa [2], Jerneja Kladnik [1], Uroš Rapuš [1], Tomaž Trobec [3], Kristina Sepčić [4], Robert Frangež [3], Ignacio E. León [2] and Iztok Turel [1,*]

[1] University of Ljubljana, Faculty of Chemistry and Chemical Technology, Večna pot 113, SI-1000 Ljubljana, Slovenia

[2] CEQUINOR (UNLP, CCT-CONICET La Plata, Asociado a CIC), Departamento de Química, Facultad de Ciencias Exactas, Universidad Nacional de La Plata. Blvd. 120 N°1465, La Plata 1900, Argentina

[3] University of Ljubljana, Veterinary Faculty, Institute of Preclinical Sciences, Gerbičeva 60, 1000 Ljubljana, Slovenia.

[4] University of Ljubljana, Biotechnical Faculty, Department of Biology, Jamnikarjeva 101, 1000 Ljubljana, Slovenia

Supporting information file

Abstract: Organoruthenium pyrithione (1-hydroxypyridine-2-thione) complexes have been shown in our recent studies to be a promising family of compounds for the development of new anticancer drugs. The complex [(η^6-*p*-cymene)Ru(pyrithionato)(pta)]PF_6 contains the phosphine ligand pta (1,3,5-triaza-7-phosphaadamantane) as a functionality that improves the stability of the complex and its aqueous solubility. Here we report our efforts to find pta alternatives and discover new structural elements to improve the biological properties of ruthenium anticancer drugs. The pta ligand was replaced by a selection of phosphine, phosphite, and arsine ligands to identify new functionalities leading to an improvement in inhibitory potency towards the enzyme glutathione *S*-transferase. In addition, cytotoxicity in breast, bone and colon cancers was investigated.

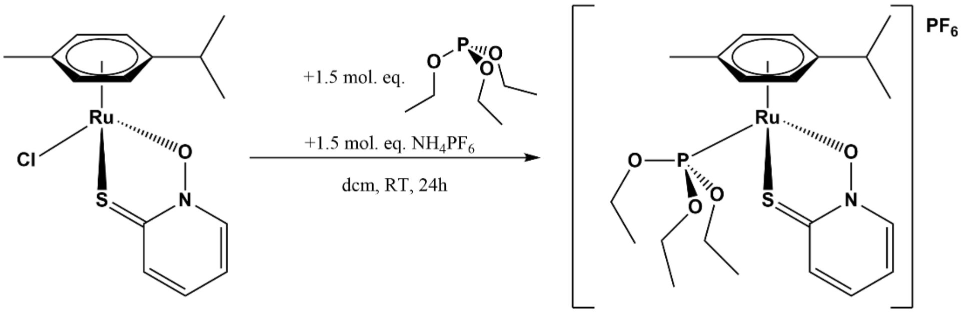

Figure S1: Synthesis of complex **3**.

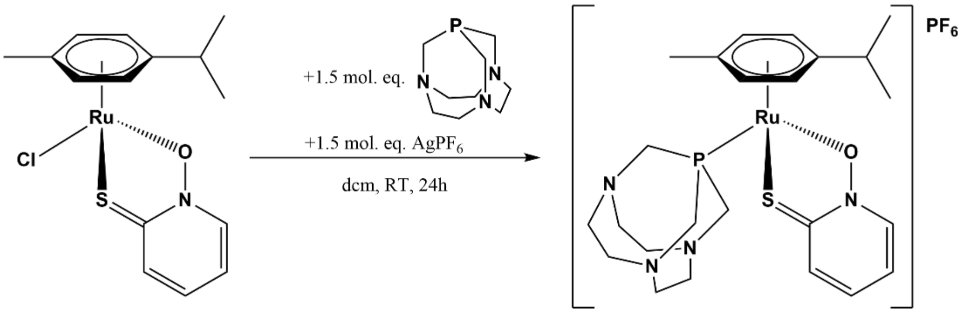

Figure S2: Synthesis of complex **4**.

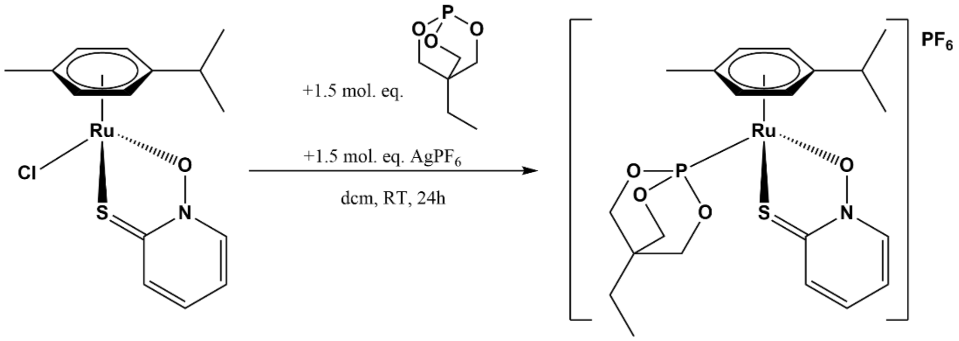

Figure S3: Synthesis of complex **5**.

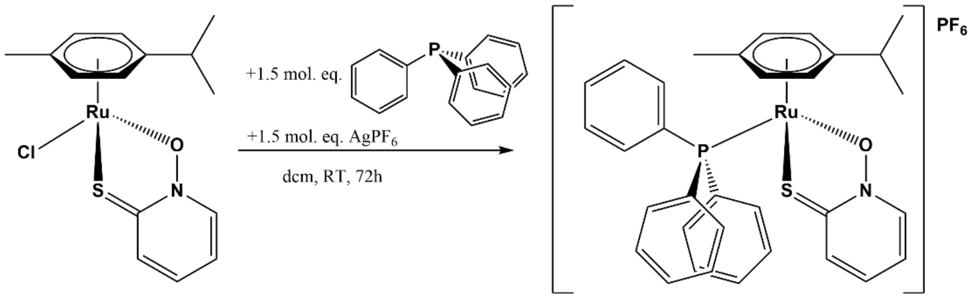

Figure S4: Synthesis of complex **6**.

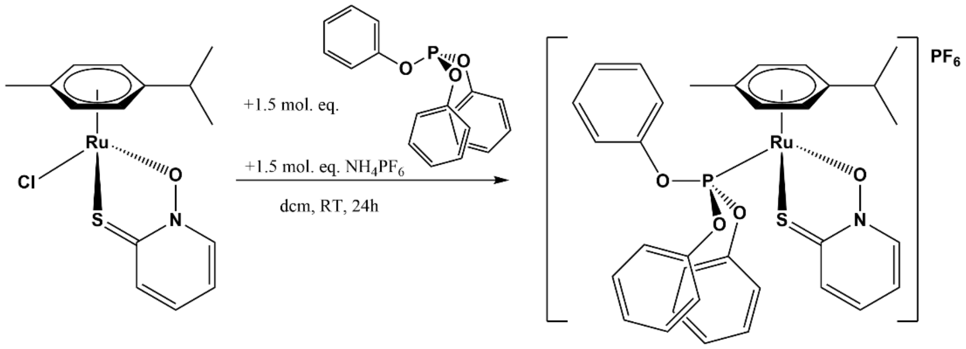

Figure S5: Synthesis of complex **7**.

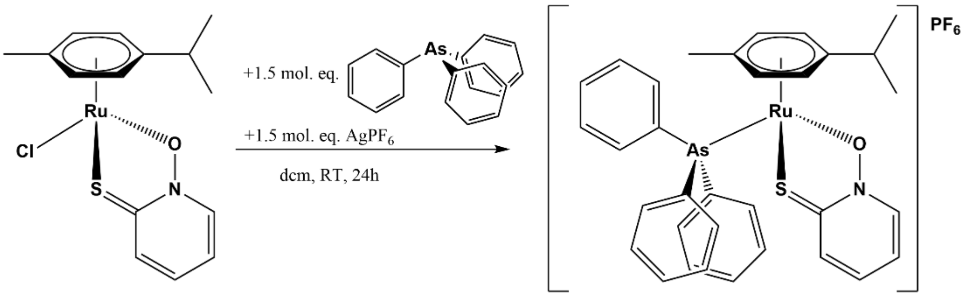

Figure S6: Synthesis of complex **8**.

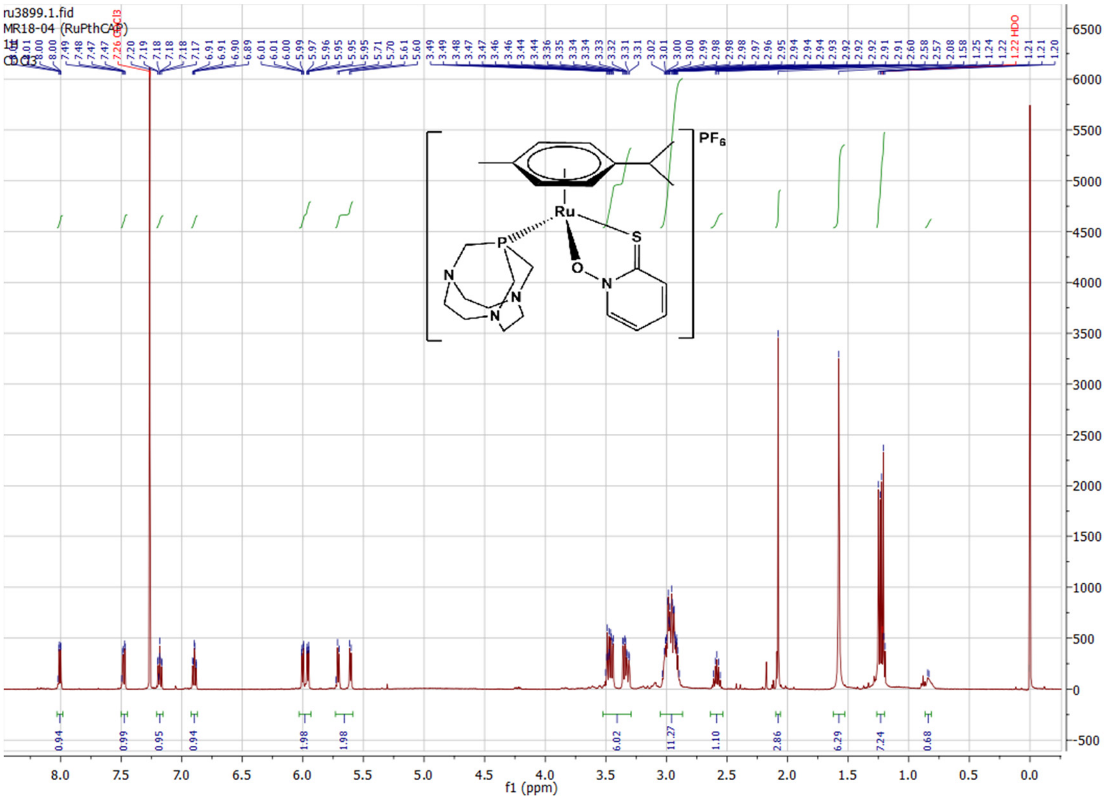

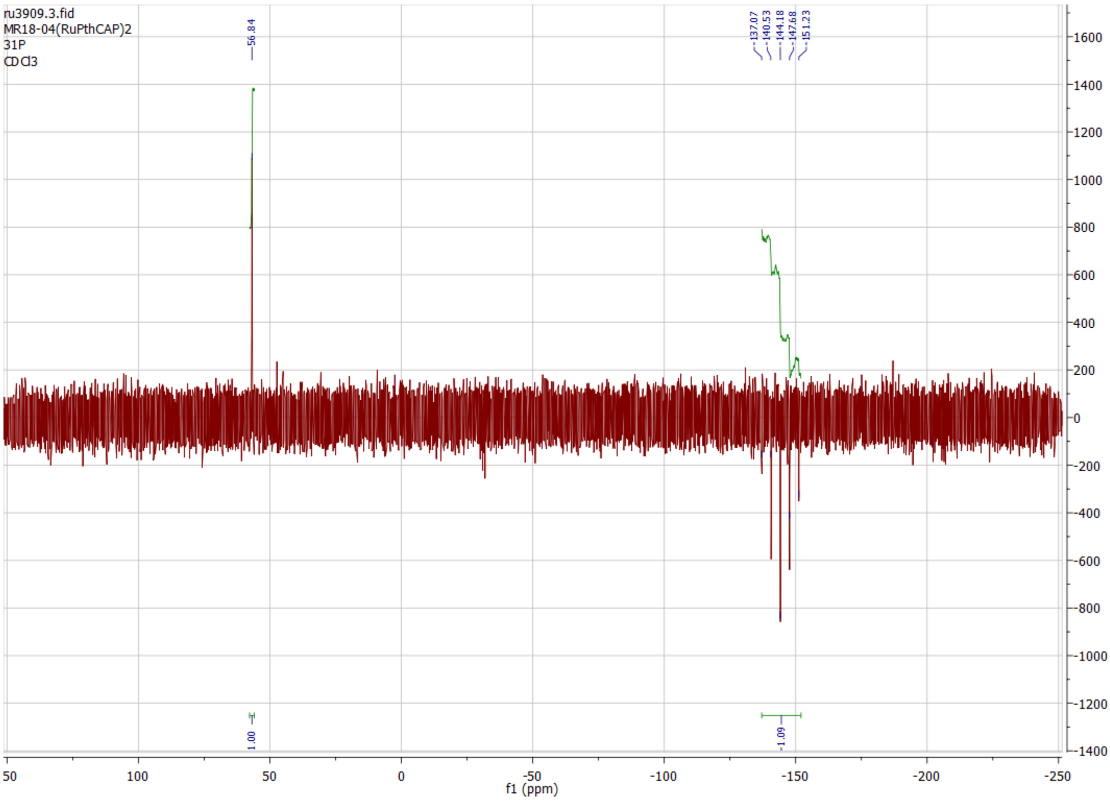

Figure S7: ^{1}H and ^{31}P NMR spectra of complex **3**.

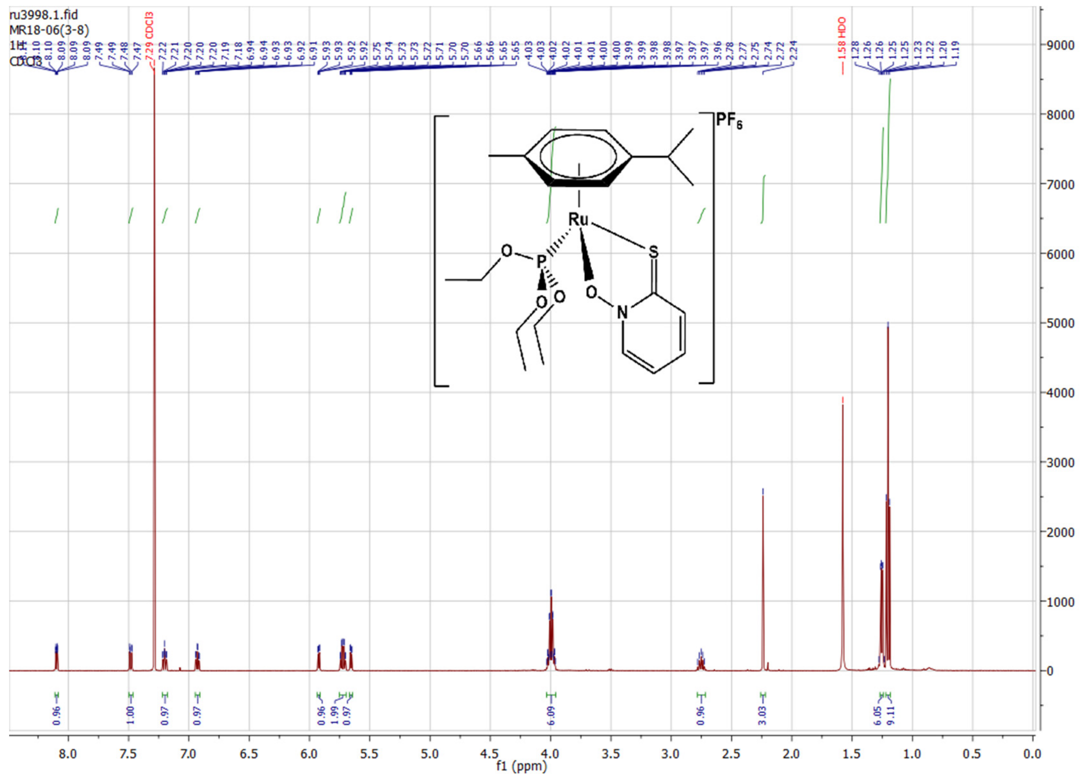

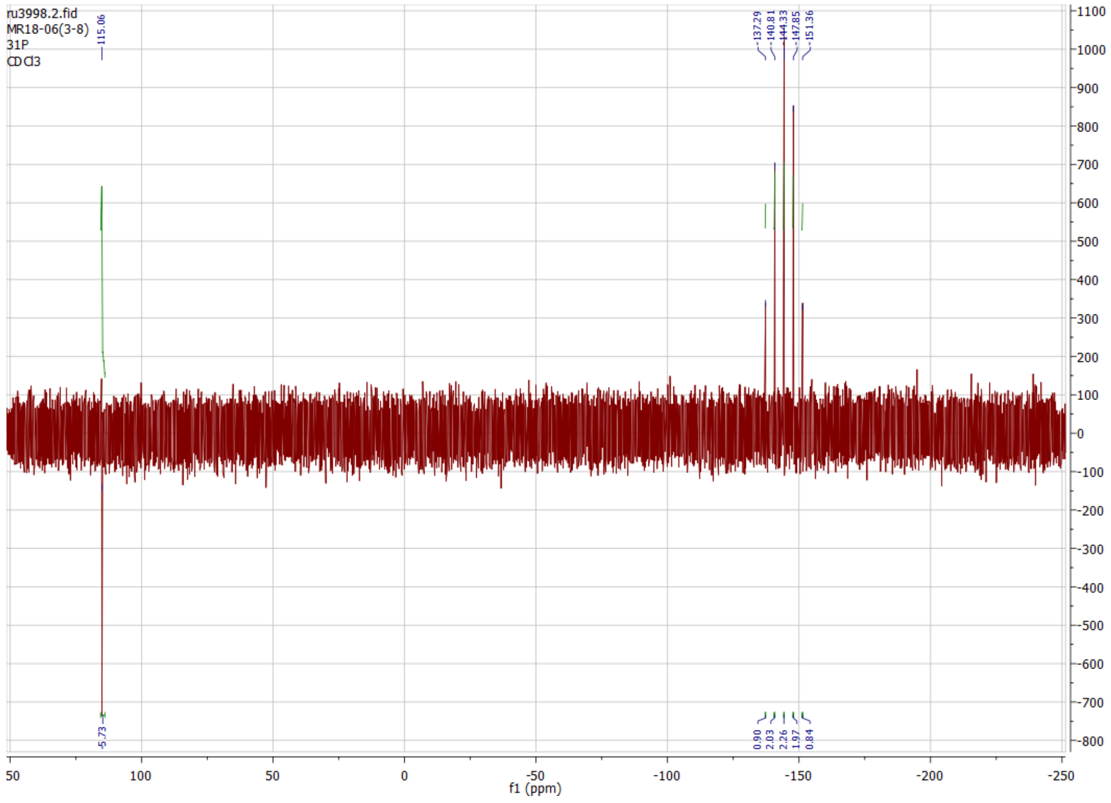

Figure S8: ^{1}H and ^{31}P NMR spectra of complex **4**.

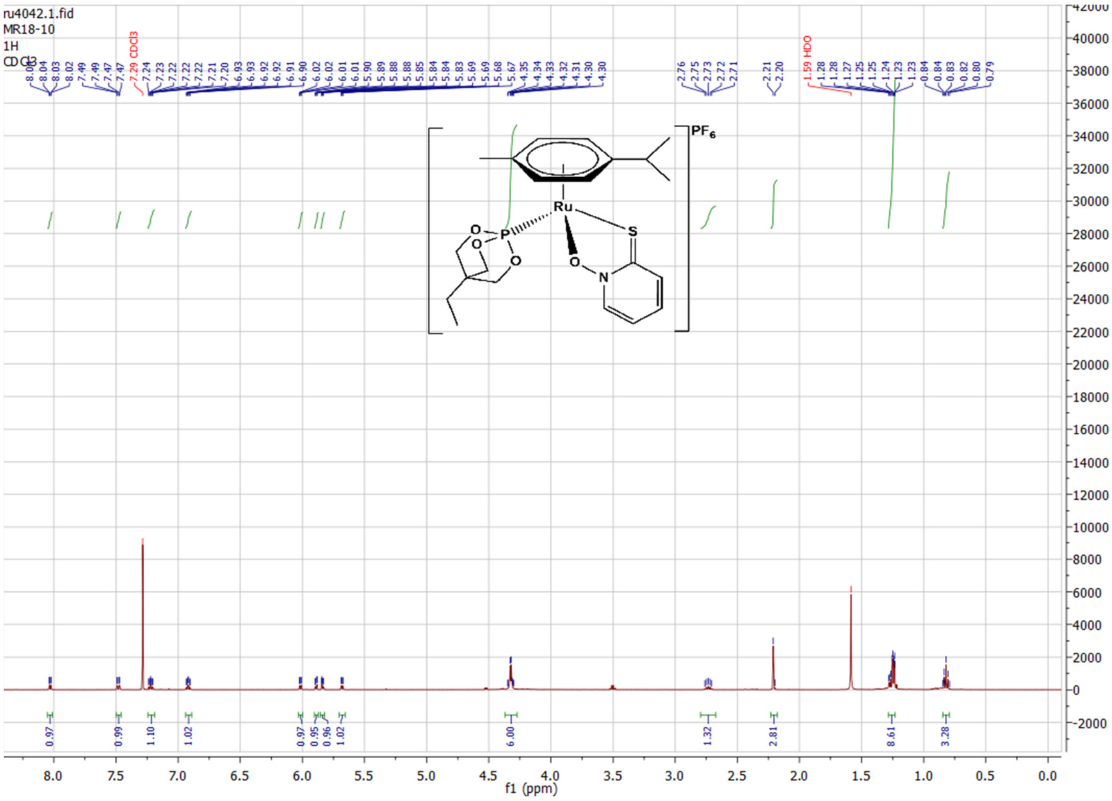

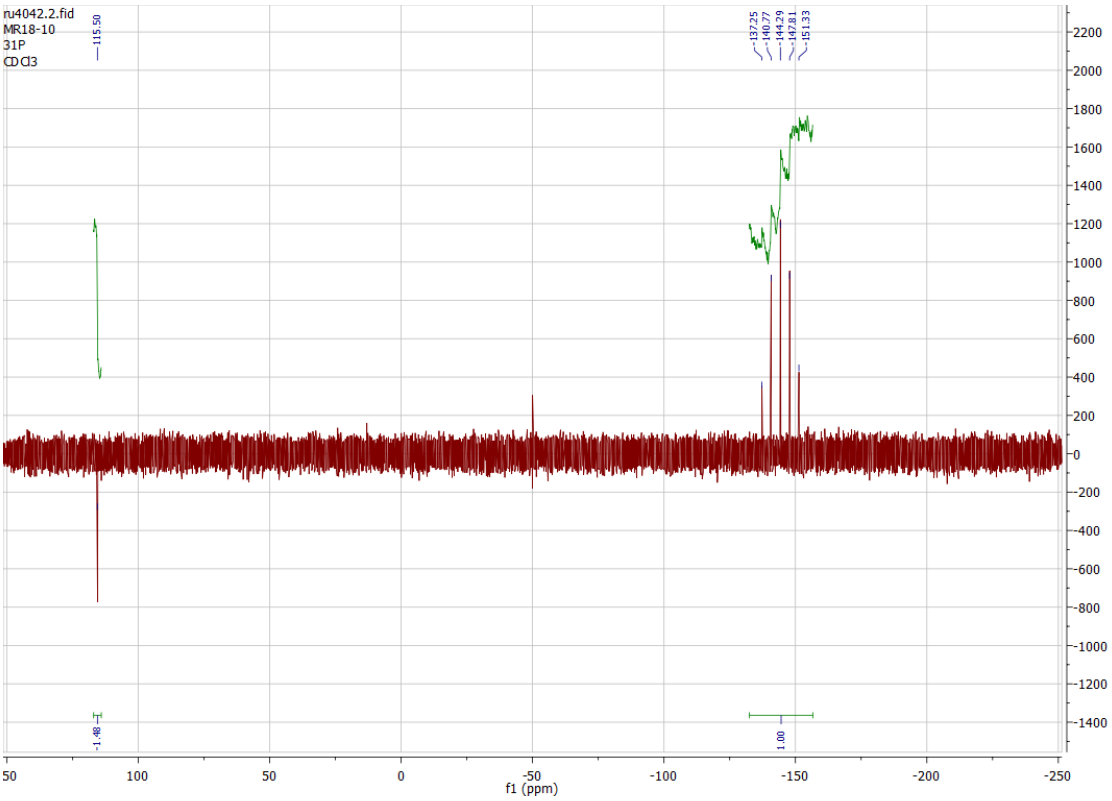

Figure S9: ^{1}H and ^{31}P NMR spectra of complex **5**.

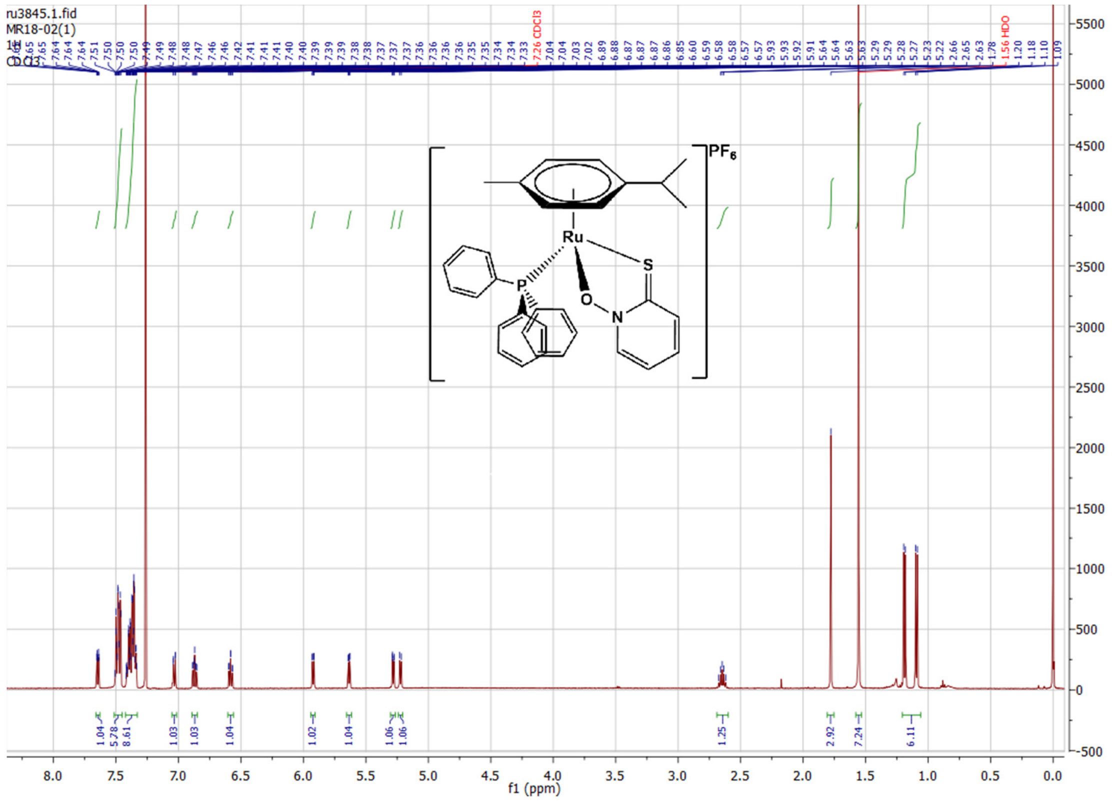

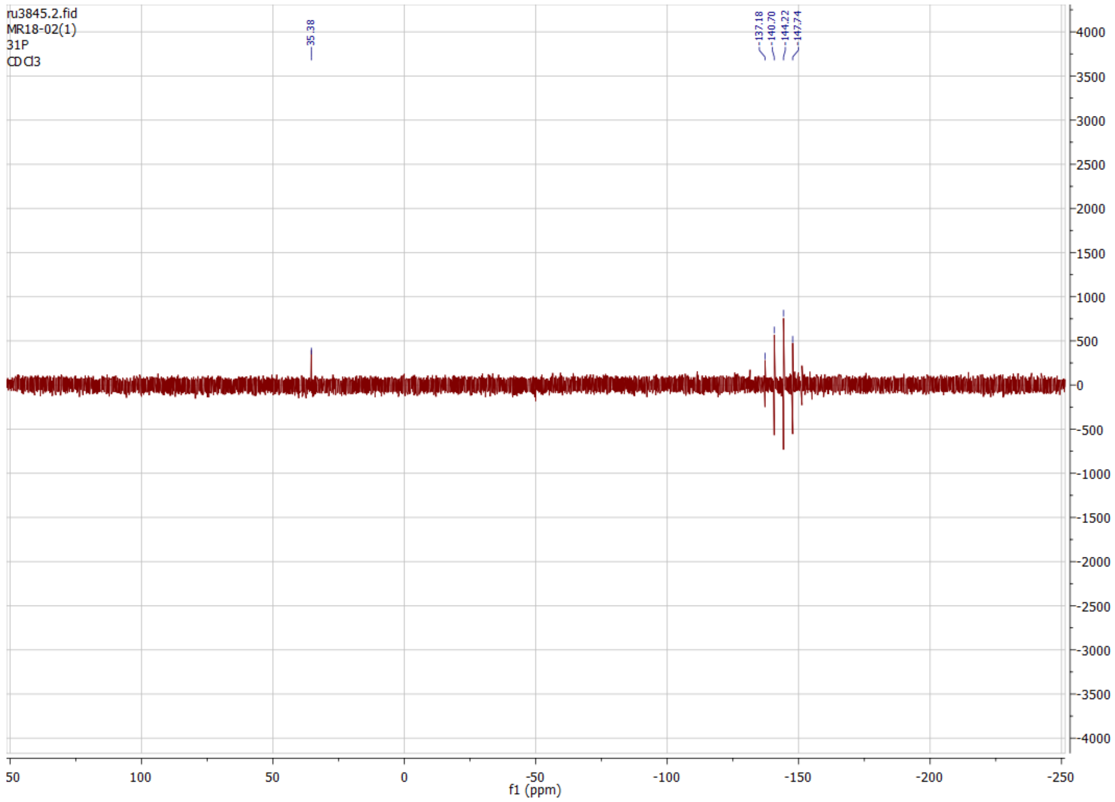

Figure S10: ${}^{1}H$ and ${}^{31}P$ NMR spectra of complex **6**.

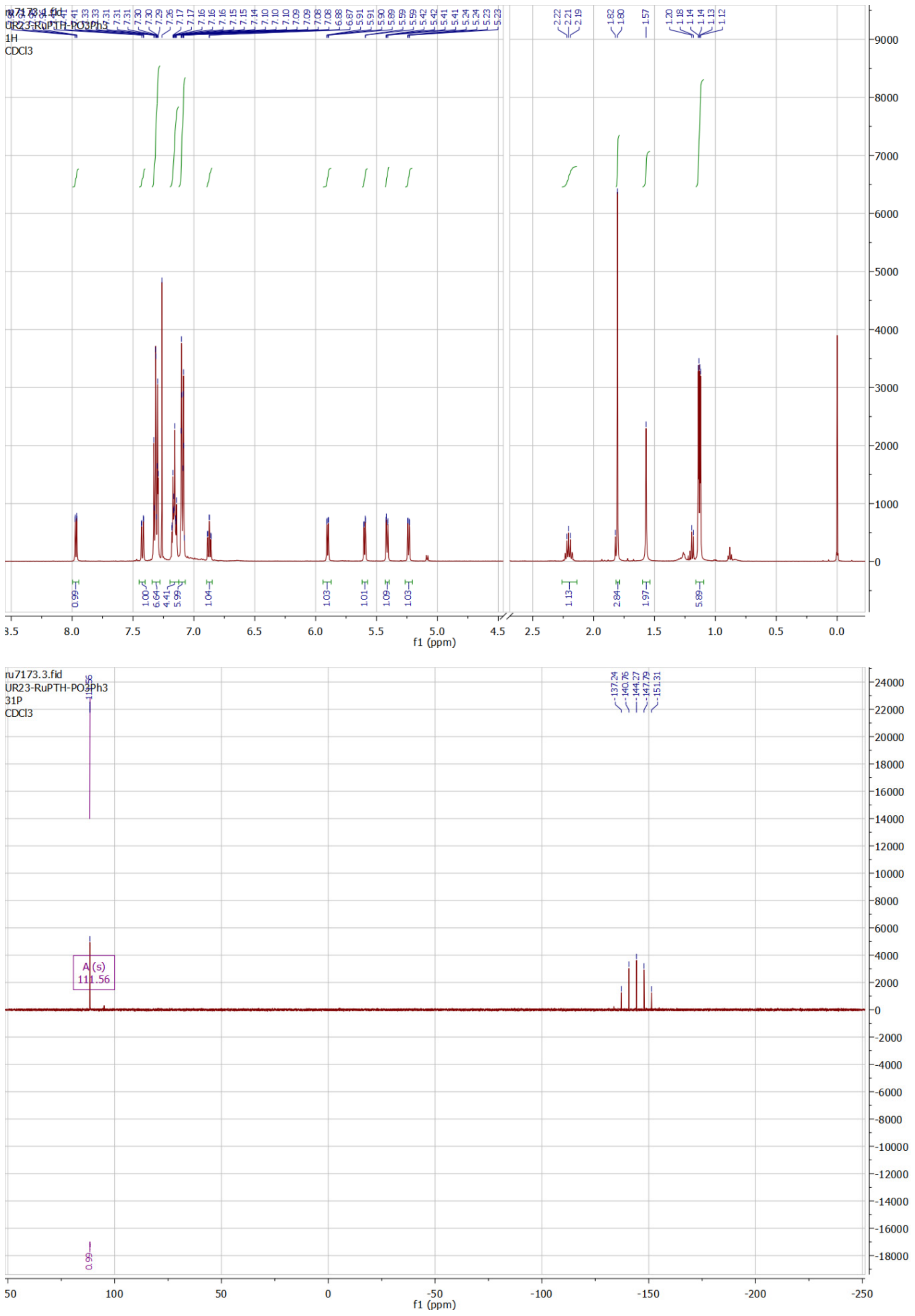

Figure S11 Figure S12: ^{1}H and ^{31}P NMR spectra of complex **7**.

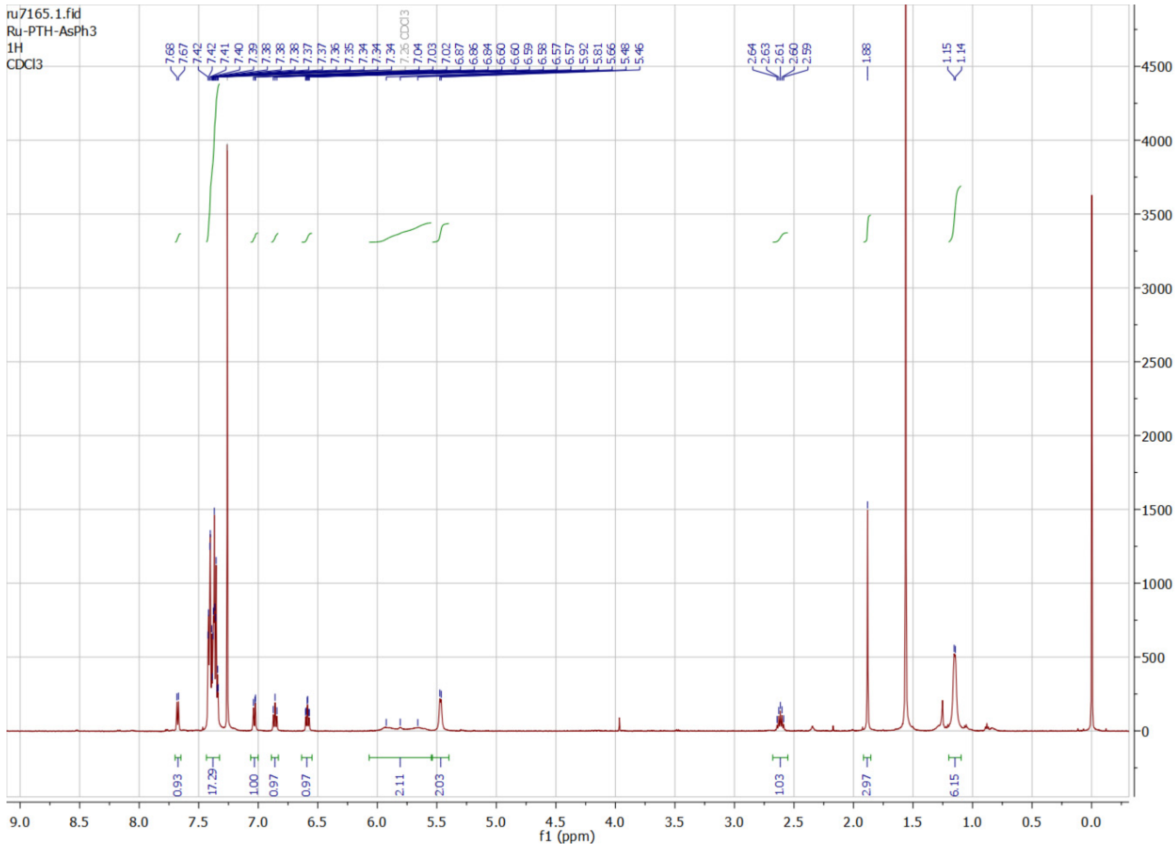

Figure S13: ^{1}H spectrum of complex **8**.

Table S1: Crystalographic data:

Compound	**6**	**8**
CCDC No.	2235957	2235958
Empirical formula	$C_{33}H_{33}F_6NOP_2RuS$	$C_{33}H_{33}AsF_6NOPRuS$
Formula weight	768.67	812.62
Temperature/K	150.00(10)	150.00(10)
Crystal system	monoclinic	monoclinic
Space group	$P2_1/c$	$P2_1/c$
a/Å	9.8473(3)	9.8252(4)
b/Å	15.5924(7)	15.7543(6)
c/Å	20.5046(7)	20.4822(8)
α/°	90	90
β/°	91.508(3)	92.506(4)
γ/°	90	90
Volume/Å^3	3147.2(2)	3167.4(2)
Z	4	4
ρ_{calc} g/cm^3	1.622	1.704
μ/mm^{-1}	0.731	1.713
F(000)	1560.0	1632.0
Crystal size/mm^3	0.3 × 0.2 × 0.2	0.15 × 0.15 × 0.05
Radiation	Mo Kα (λ = 0.71073)	Mo Kα (λ = 0.71073)
2Θ range for data collection/°	5.814 to 54.97	4.89 to 54.968
Index ranges	$-6 \leq h \leq 12$, $-20 \leq k \leq 19$, $-26 \leq l \leq 26$	$-12 \leq h \leq 12$, $-20 \leq k \leq 14$, $-26 \leq l \leq 24$
Reflections collected	16965	14677
Independent reflections	7206 [R_{int} = 0.0334, R_{sigma} = 0.0511]	7242 [R_{int} = 0.0314, R_{sigma} = 0.0460]
Data/restraints/parameters	7206/0/409	7242/0/409
Goodness-of-fit on F^2	1.030	1.028
Final R indexes [I ≥ 2σ (I)]	R_1 = 0.0430, wR_2 = 0.0991	R_1 = 0.0389, wR_2 = 0.0900
Final R indexes [all data]	R_1 = 0.0634, wR_2 = 0.1077	R_1 = 0.0506, wR_2 = 0.0968
Largest diff. peak/hole / e/Å^3	1.67/-0.91	1.52/-0.78
Flack parameter	/	/

Figure S14: Photographs of single crystal samples of **6** and **8**:

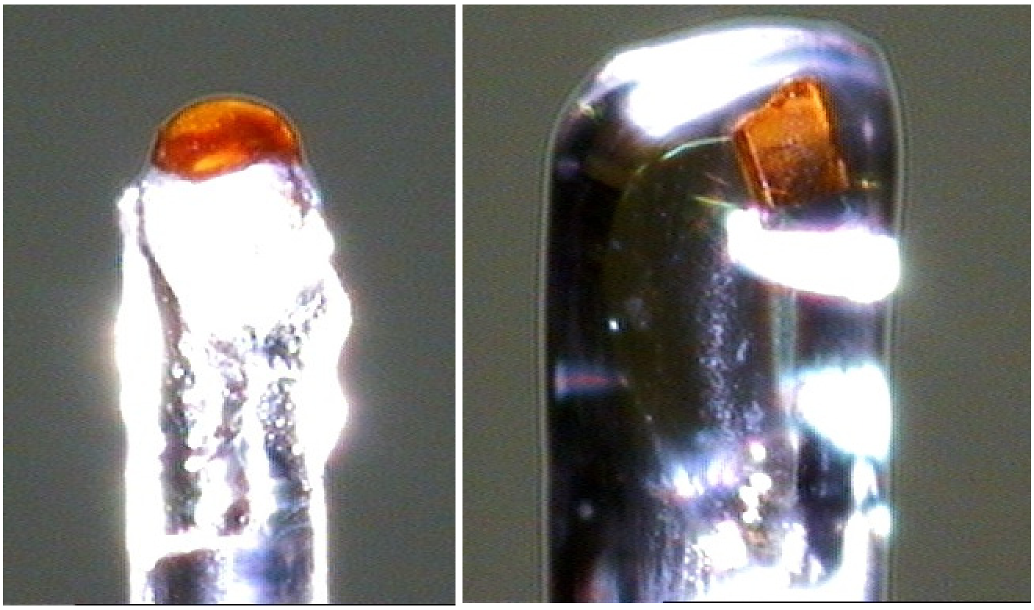

Supplement: Supplementary file 1 [file molecules-28-02499-s001.zip › molecules-2221733-supplementary.pdf]
